# Supplementary material for: Evidence for DNA-mediated nuclear compartmentalization distinct from phase separation
Source: eLife. 2019 May 7;8:e47098. doi: 10.7554/eLife.47098 (PMC6522219; doi:10.7554/eLife.47098)
Supplement: Figure 1—source data 1. — Each protein listed was analyzed as described in the Materials and methods section, and regions with an IUPred score of greater than 0.55 were recorded. [file elife-47098-fig1-data1.docx]

| **gene name** | **function** | **Class** | **Protein Length** | **Region Start** | **Region End** | **IDR Length** |
| --- | --- | --- | --- | --- | --- | --- |
| RS1 | transcription regulation | alpha | 1294 | 0 | 79 | 79 |
|  |  |  |  | 94 | 273 | 179 |
|  |  |  |  | 594 | 615 | 21 |
|  |  |  |  | 717 | 820 | 103 |
|  |  |  |  | 884 | 893 | 9 |
|  |  |  |  | 1214 | 1236 | 22 |
|  |  |  |  | 1276 | 1294 | 18 |
| US12 | transcription regulation | alpha | 88 | 35 | 88 | 53 |
| US11 | tegument | tegument | 149 | 0 | 149 | 149 |
| US10 | tegument | tegument | 300 | 23 | 92 | 69 |
|  |  |  |  | 124 | 156 | 32 |
| US9 | membrane/glycoprotein | beta | 57 | 0 | 16 | 16 |
|  |  |  |  | 54 | 57 | 3 |
| US8 | membrane/glycoprotein | beta | 190 | 0 | 5 | 5 |
|  |  |  |  | 31 | 104 | 73 |
|  |  |  |  | 172 | 190 | 18 |
| US8A | membrane/glycoprotein | beta | 550 | 161 | 215 | 54 |
|  |  |  |  | 392 | 410 | 18 |
|  |  |  |  | 480 | 550 | 70 |
| US7 | membrane/glycoprotein | beta | 383 | 195 | 253 | 58 |
|  |  |  |  | 325 | 383 | 58 |
| US6 | membrane/glycoprotein | beta | 394 | 277 | 319 | 42 |
|  |  |  |  | 381 | 394 | 13 |
| US4 | membrane/glycoprotein | gamma | 239 | 33 | 170 | 137 |
| US3 | kinase/phosphatase | beta | 481 | 19 | 170 | 151 |
| US2 | unknown | gamma | 291 | 254 | 291 | 37 |
| US1.5 | unknown | alpha | 250 | 0 | 10 | 10 |
|  |  |  |  | 137 | 212 | 75 |
| US1 | replication | alpha | 420 | 0 | 14 | 14 |
|  |  |  |  | 15 | 181 | 166 |
|  |  |  |  | 307 | 382 | 75 |
| RL2 | transcription regulation | alpha | 776 | 0 | 106 | 106 |
|  |  |  |  | 222 | 568 | 346 |
|  |  |  |  | 595 | 628 | 33 |
|  |  |  |  | 761 | 776 | 15 |
| UL56 | membrane/glycoprotein | beta | 234 | 0 | 101 | 101 |
|  |  |  |  | 151 | 187 | 36 |
| UL55 | unknown | beta | 186 | 179 | 186 | 7 |
| UL54 | transcription regulation | alpha | 512 | 0 | 243 | 243 |
| UL52 | replication | beta | 1058 | 0 | 12 | 12 |
|  |  |  |  | 380 | 389 | 9 |
|  |  |  |  | 480 | 497 | 17 |
|  |  |  |  | 699 | 735 | 36 |
|  |  |  |  | 1055 | 1058 | 3 |
| UL51 | tegument | tegument | 244 | 180 | 244 | 64 |
| UL50 | replication | beta | 371 | 0 | 7 | 7 |
|  |  |  |  | 155 | 159 | 4 |
|  |  |  |  | 333 | 371 | 38 |
| UL49A | membrane/glycoprotein | beta | 91 | 29 | 44 | 15 |
| UL49 | tegument | tegument | 301 | 0 | 181 | 181 |
|  |  |  |  | 270 | 301 | 31 |
| UL48 | transcription regulation | gamma | 490 | 0 | 46 | 46 |
|  |  |  |  | 454 | 456 | 2 |
|  |  |  |  | 487 | 490 | 3 |
| UL47 | tegument | tegument | 693 | 0 | 26 | 26 |
|  |  |  |  | 51 | 125 | 74 |
|  |  |  |  | 150 | 180 | 30 |
|  |  |  |  | 680 | 693 | 13 |
| UL46 | tegument | tegument | 719 | 0 | 8 | 8 |
|  |  |  |  | 435 | 540 | 105 |
|  |  |  |  | 561 | 606 | 45 |
|  |  |  |  | 667 | 694 | 27 |
|  |  |  |  | 706 | 719 | 13 |
| UL45 | membrane/glycoprotein | gamma | 172 | 0 | 10 | 10 |
| UL44 | membrane/glycoprotein | gamma | 511 | 39 | 125 | 86 |
|  |  |  |  | 314 | 330 | 16 |
| UL43 | membrane/glycoprotein | gamma | 415 | 0 | 17 | 17 |
|  |  |  |  | 218 | 255 | 37 |
| UL42 | replication | beta | 489 | 0 | 25 | 25 |
|  |  |  |  | 333 | 448 | 115 |
|  |  |  |  | 477 | 488 | 11 |
|  |  |  |  | 110 | 148 | 38 |
| UL41 | tegument | tegument | 489 | 286 | 367 | 81 |
| UL40 | replication | beta | 340 | 0 | 13 | 13 |
| UL39 | replication | beta | 1137 | 0 | 30 | 30 |
|  |  |  |  | 125 | 157 | 32 |
|  |  |  |  | 176 | 309 | 133 |
| UL38 | capsid | gamma | 465 | 0 | 56 | 56 |
|  |  |  |  | 70 | 85 | 15 |
|  |  |  |  | 144 | 155 | 11 |
|  |  |  |  | 357 | 388 | 31 |
| UL37 | tegument | tegument | 1123 | 0 | 46 | 46 |
|  |  |  |  | 971 | 977 | 6 |
|  |  |  |  | 1057 | 1123 | 66 |
| UL36 | tegument | tegument | 3136 | 0 | 22 | 22 |
|  |  |  |  | 268 | 382 | 114 |
|  |  |  |  | 396 | 495 | 99 |
|  |  |  |  | 749 | 770 | 21 |
|  |  |  |  | 948 | 971 | 23 |
|  |  |  |  | 1254 | 1282 | 28 |
|  |  |  |  | 1911 | 1925 | 14 |
|  |  |  |  | 2267 | 2291 | 24 |
|  |  |  |  | 2489 | 2534 | 45 |
|  |  |  |  | 2553 | 2701 | 148 |
|  |  |  |  | 2728 | 2984 | 256 |
|  |  |  |  | 3029 | 3066 | 37 |
| UL35 | capsid | gamma | 112 | 0 | 10 | 10 |
|  |  |  |  | 40 | 46 | 6 |
|  |  |  |  | 103 | 112 | 9 |
| UL34 | membrane/glycoprotein | gamma | 275 | 0 | 11 | 11 |
| UL33 | packaging | gamma | 130 | 0 | 14 | 14 |
| UL32 | packaging | gamma | 596 | 0 | 5 | 5 |
|  |  |  |  | 77 | 107 | 30 |
|  |  |  |  | 227 | 237 | 10 |
| UL31 | other | gamma | 306 | 0 | 41 | 41 |
| UL30 | replication | beta | 1235 | 0 | 16 | 16 |
|  |  |  |  | 50 | 61 | 11 |
|  |  |  |  | 644 | 693 | 49 |
|  |  |  |  | 1099 | 1134 | 35 |
|  |  |  |  | 1233 | 1235 | 2 |
| UL29 | replication | beta | 1196 | 0 | 8 | 8 |
|  |  |  |  | 288 | 307 | 19 |
|  |  |  |  | 1158 | 1196 | 38 |
| UL28 | packaging | gamma | 785 | 265 | 288 | 23 |
|  |  |  |  | 435 | 491 | 56 |
|  |  |  |  | 778 | 785 | 7 |
| UL27 | membrane/glycoprotein | beta | 904 | 45 | 99 | 54 |
|  |  |  |  | 469 | 492 | 23 |
|  |  |  |  | 819 | 836 | 17 |
|  |  |  |  | 881 | 904 | 23 |
| UL26.5 | capsid | gamma | 329 | 0 | 15 | 15 |
|  |  |  |  | 108 | 171 | 63 |
|  |  |  |  | 211 | 295 | 84 |
| UL26 | capsid | gamma | 635 | 0 | 11 | 11 |
|  |  |  |  | 270 | 318 | 48 |
|  |  |  |  | 414 | 477 | 63 |
|  |  |  |  | 517 | 601 | 84 |
| UL25 | packaging | gamma | 580 | 108 | 132 | 24 |
| UL24 | unknown | gamma | 269 | 0 | 3 | 3 |
|  |  |  |  | 190 | 242 | 52 |
|  |  |  |  | 265 | 269 | 4 |
| UL23 | replication | beta | 376 | 0 | 41 | 41 |
|  |  |  |  | 263 | 280 | 17 |
|  |  |  |  | 371 | 376 | 5 |
| UL22 | membrane/glycoprotein | gamma | 838 | 175 | 210 | 35 |
|  |  |  |  | 293 | 317 | 24 |
| UL21 | tegument | tegument | 535 | 206 | 231 | 25 |
|  |  |  |  | 246 | 277 | 31 |
| UL20 | membrane/glycoprotein | gamma | 222 | 0 | 10 | 10 |
| UL19 | capsid | gamma | 1374 | 0 | 7 | 7 |
| UL18 | capsid | gamma | 318 | 0 | 2 | 2 |
| UL17 | packaging | gamma | 703 | 202 | 250 | 48 |
| UL16 | tegument | tegument | 373 | 0 | 32 | 32 |
|  |  |  | 373 | 154 | 175 | 21 |
| UL15 | packaging | gamma | 735 | 0 | 3 | 3 |
| UL14 | tegument | tegument | 219 | 0 | 7 | 7 |
|  |  |  |  | 164 | 219 | 55 |
| UL13 | tegument | tegument | 518 | 0 | 115 | 115 |
| UL12 | replication | beta | 626 | 0 | 125 | 125 |
|  |  |  |  | 603 | 626 | 23 |
| UL11 | tegument | tegument | 96 | 48 | 96 | 48 |
| UL10 | membrane/glycoprotein | gamma | 473 | 0 | 14 | 14 |
|  |  |  |  | 366 | 427 | 61 |
|  |  |  |  | 444 | 473 | 29 |
| UL9 | replication | beta | 851 | 0 | 19 | 19 |
|  |  |  |  | 263 | 283 | 20 |
| UL7 | tegument | tegument | 296 | 0 | 6 | 6 |
| UL6 | capsid | gamma | 676 | 0 | 17 | 17 |
|  |  |  |  | 384 | 401 | 17 |
|  |  |  |  | 629 | 676 | 47 |
| UL5 | replication | beta | 882 | 0 | 34 | 34 |
|  |  |  |  | 602 | 629 | 27 |
| UL4 | unknown | gamma | 199 | 149 | 160 | 11 |
| UL3 | unknown | gamma | 235 | 42 | 88 | 46 |
|  |  |  |  | 144 | 158 | 14 |
|  |  |  |  | 233 | 235 | 2 |
| UL2 | DNA repair | beta | 334 | 0 | 94 | 94 |
| UL1 | membrane/glycoprotein | gamma | 224 | 164 | 224 | 60 |
| RL1 | transcription regulation | late | 240 | 0 | 130 | 130 |
|  |  |  |  | 144 | 168 | 24 |
|  |  |  |  | 236 | 240 | 4 |
| LRP1 | latency | N/A | 300 | 0 | 99 | 99 |
|  |  |  |  | 256 | 300 | 44 |
| LRP2 | latency | N/A | 60 | 0 | 60 | 60 |
|  | | | | | | |
