# Supplementary material for: Evidence for DNA-mediated nuclear compartmentalization distinct from phase separation
Source: eLife. 2019 May 7;8:e47098. doi: 10.7554/eLife.47098 (PMC6522219; doi:10.7554/eLife.47098)
Supplement: Figure 1—source data 2. — Gene name, organism of origin, size, and the fraction of the protein that scores as an IDR according to the analysis described in the Materials and methods section. References and the citation within and provided. [file elife-47098-fig1-data2.docx]

| **Protein** | **Organism^a^** | **Protein Length (AA)** | **Total IDR length (AA)** | **IDR Fraction** | **Reference^b^** |
| --- | --- | --- | --- | --- | --- |
| FUS | Hs | 526 | 397 | 0.75475285 | Chong et al., 2018 |
| Taf15 | Hs | 592 | 274 | 0.46283784 |  |
| EWS | Hs | 656 | 420 | 0.6402439 |  |
| SP1 | Hs | 785 | 220 | 0.28025478 |  |
| Huntingtin | Hs | 3142 | 311 | 0.09898154 | Bergeron-Sandoval et al., 2016 |
| PML | Hs | 882 | 145 | 0.16439909 |  |
| PGL-1 | Ce | 730 | 94 | 0.12876712 |  |
| RPB1 | Hs | 1970 | 119 | 0.06040609 | Boehning et al., 2018 |
| DDX4 | Hs | 724 | 144 | 0.19889503 | Courchaine et al., 2016 |
| eIF4GII | Sc | 907 | 229 | 0.25248071 |  |
| Fibrillarin | Hs | 321 | 89 | 0.27725857 |  |
| hnRNPA1 | Hs | 320 | 48 | 0.15 |  |
| Laf1 | Ce | 708 | 232 | 0.32768362 |  |
| Lsm4 | Sc | 187 | 92 | 0.49197861 |  |
| RBM14 | Hs | 669 | 85 | 0.12705531 |  |
| SRSF2 | Hs | 221 | 135 | 0.61085973 |  |
| TDP-43 | Hs | 414 | 82 | 0.19806763 |  |
| Tia1 | Hs | 386 | 35 | 0.09067358 |  |
| Whi3 | Ag | 729 | 359 | 0.49245542 |  |
| PUB1 | Hs | 453 | 213 | 0.47019868 |  |
| HP1a | Dm | 213 | 68 | 0.31924883 | Strom et al., 2017 |
| DAXX | Hs | 740 | 407 | 0.55 | Banani et al., 2017 |
| PGL-3 | Ce | 693 | 124 | 0.17893218 |  |
| NPM1 | Hs | 294 | 125 | 0.42517007 |  |
| hRNPAB | Hs | 332 | 93 | 0.28012048 | Aguzzi and Altmeyer, 2016 |
| hnRNPA3 | Hs | 378 | 56 | 0.14814815 |  |
| hnRNPA2B1 | Hs | 353 | 41 | 0.11614731 |  |
| hnRNPD | Hs | 355 | 40 | 0.11267606 |  |
| hnRDL | Hs | 420 | 127 | 0.30238095 |  |
| NUP145 | Sc | 1317 | 219 | 0.16628702 | Schmidt and Görlich, 2015 |
| a) Organism abbreviations: Hs, Homo sapiens; Ce, Caenorhabditis elegans; Sc, Saccharomyces cerevisiae; Dm, Drosophila melanogaster; Ag, Ashbya gossypii b) May include citations within reference. | | | | | |
